# Comparing the coverage, recall, and precision of searches for 120 systematic reviews in Embase, MEDLINE, and Google Scholar: a prospective study

**DOI:** 10.1186/s13643-016-0215-7

**Published:** 2016-03-01

**Authors:** Wichor M. Bramer, Dean Giustini, Bianca M. R. Kramer

**Affiliations:** Erasmus MC, University Medical Center Rotterdam, Medical Library, PO Box 2040, 3000 CA Rotterdam, The Netherlands; The University of British Columbia, UBC Biomedical Branch Library, Gordon and Leslie Diamond Health Care Centre, 2775 Laurel Street, Floor 2, Vancouver, BC V5Z 1 M9 Canada; Utrecht University Library, PO Box 80125, 3508 TC Utrecht, The Netherlands

**Keywords:** Information storage and retrieval, Review literature as topic, Bibliographic databases, Search engine, Sensitivity and specificity

## Abstract

**Background:**

Previously, we reported on the low recall of Google Scholar (GS) for systematic review (SR) searching. Here, we test our conclusions further in a prospective study by comparing the coverage, recall, and precision of SR search strategies previously performed in Embase, MEDLINE, and GS.

**Methods:**

The original search results from Embase and MEDLINE and the first 1000 results of GS for librarian-mediated SR searches were recorded. Once the inclusion-exclusion process for the resulting SR was complete, search results from all three databases were screened for the SR’s included references. All three databases were then searched post hoc for included references not found in the original search results.

**Results:**

We checked 4795 included references from 120 SRs against the original search results. Coverage of GS was high (97.2 %) but marginally lower than Embase and MEDLINE combined (97.5 %). MEDLINE on its own achieved 92.3 % coverage. Total recall of Embase/MEDLINE combined was 81.6 % for all included references, compared to GS at 72.8 % and MEDLINE alone at 72.6 %. However, only 46.4 % of the included references were among the downloadable first 1000 references in GS. When examining data for each SR, the traditional databases’ recall was better than GS, even when taking into account included references listed beyond the first 1000 search results. Finally, precision of the first 1000 references of GS is comparable to searches in Embase and MEDLINE combined.

**Conclusions:**

Although overall coverage and recall of GS are high for many searches, the database does not achieve full coverage as some researchers found in previous research. Further, being able to view only the first 1000 records in GS severely reduces its recall percentages. If GS would enable the browsing of records beyond the first 1000, its recall would increase but not sufficiently to be used alone in SR searching. Time needed to screen results would also increase considerably. These results support our assertion that neither GS nor one of the other databases investigated, is on its own, an acceptable database to support systematic review searching.

**Electronic supplementary material:**

The online version of this article (doi:10.1186/s13643-016-0215-7) contains supplementary material, which is available to authorized users.

## Background

In 2013, an article by Gehanno et al. [1] prompted a discussion around the utility of Google Scholar (GS) to support systematic review (SR) searching. In response, we examined the recall of GS and PubMed search strategies for included references of published biomedical SRs [2]. There, we determined that the recall of all included references found among the first 1000 search results in GS was insufficient for it to be used on its own to support SR searching.

In our 2013 study, we intentionally selected search strategies that were identical in PubMed and GS, in an effort to study the effect of the database, instead of the quality of the query translation. Therefore, the search strategies used for PubMed in our previous study did not fully use the possibilities of a traditional database search strategy. Librarian-mediated searches (combining MeSH terms and free text terms in traditional databases) achieve better results than non-librarian searches [3, 4]. References that would have been included, had they been retrieved, could have been missed in PubMed due to a lack of MeSH terms in the search strategies. In our previous paper, we showed that the optimization of search strategies in PubMed (adding MeSH terms and more synonyms) led to more improvement than a similar process in GS (adding extra terms found in the included references). Here, we investigate whether an experienced information specialist, an expert at performing systematic review searches, can find all included references using only one database.

Our prior research replicated search strategies used in previously published systematic reviews. One of GS’ shortcomings is that searches are never wholly replicable later, as the search algorithm is constantly changing day to day. GS can only limit search results to publication date ranges. In traditional databases such as PubMed, search results can be limited to specific dates, such as MeSH date (date when MeSH terms were altered), or entry date (date a record was added to database). Search results can be reproduced in PubMed as they were performed on a specific day, month, and year.

GS does not only index papers which it found as full text but also find references merely because they were cited by papers (which are then marked as [citation]). In this article, we refer to references marked by GS as [citation] as “citation only.” As we used only published SRs in our previous paper, for which most of the full text had already been indexed by GS, we hypothesize that GS probably covered all included references at least as citation only. Due to GS’s ever-changing database, search engine, and relevance ranking algorithm, searchers are never confident these *citation only* results were present at the time of the original search.

As a follow-up, we aim to evaluate the search results of systematic review search strategies created by an experienced information specialist at the time they were conducted in MEDLINE via the Ovid interface, combined searching in Embase and MEDLINE via Embase.com and GS. Our goal is to compare the coverage of these databases and their performance in terms of precision and recall for included references in SRs.

## Methods

The first author regularly performs librarian-mediated searches to support SRs in the academic hospital setting in which he works. The reviews generally cover a wide range of medical topics, from therapeutic effectiveness and diagnostic accuracy to ethics and public health. The methods used at Erasmus MC to create systematic review search strategies will be described in detail in a separate paper.

In short, the first author performs single-line search strategies in Embase.com, which are developed using a unique optimization method. The Embase.com search strategies are translated into other databases and interfaces using macros. These macros are developed in MS Word to search for syntax from one interface and replace it with an appropriate syntax for another interface. After automatic translation of syntax from Embase.com to MEDLINE in Ovid, Emtree terms for Embase are manually replaced with appropriate MeSH terms.

Search strategies for GS are derived from an array of words searched in titles and abstracts in Embase.com. All relevant search terms are copied, and truncated terms are expanded to the most common term(s). To adhere to the limitation of 256 characters, the length of each search strategy is reduced by replacing all Boolean operators *OR*, including its surrounding spaces with |, effectively reducing the number of characters per synonym by three. Proximity operators in Embase.com are replaced in GS by combining optional search terms in quoted phrases. Thus, if an Embase.com search strategy for *liver cancer* contains ((liver OR hepatic) NEAR/3 (cancer* OR tumor* OR neoplasm*)), this is translated to *"liver|hepatic cancer|tumor|neoplasms"* in GS. If the total number of characters in the GS search exceeds 256, the information specialist (often together with the reviewer) decides which search terms are likely to be least relevant and deletes them one at a time, until the threshold is reached. In the Additional file 1 some examples of search strategy translations between the three databases mentioned are provided.

SR searches were documented at the institution of the first author before researchers began to screen articles for inclusion. The total search results from two major biomedical databases: MEDLINE in the Ovid interface, Embase at Embase.com (searching both Embase and MEDLINE records) and Google Scholar (where Publish or Perish software [5] allowed downloading of the first 1000 search results) were imported into EndNote after searching was concluded (at the start of the systematic review project).

Reviewers obtained full search results from all databases which additionally to the aforementioned databases involved at least the Cochrane Registry of Trials, Web of Science, and a subset of PubMed to find recent articles. Occasionally, additional databases were used such as Scopus, CINAHL (via EBSCOhost), or PsycINFO (via Ovid). Reviewers were advised to seek other sources of included references by using cited and citing references tracking, contacting key authors in the field, and hand-searching journals, but the decision to do so was up to the researchers. In the first author’s institution, as in many other institutes, these tasks are generally performed by the researchers, not as a library service.

After the process of collecting included references was completed, reviewers provided us with a list of included references. Alternatively, these were retrieved using the reference lists of resulting publications. We searched for all included references one-by-one in the original files in EndNote, using author names, year, and if necessary parts of the title. Record numbers of positive matches in EndNote were used to determine the database(s) from which each included reference was retrieved.

For included references not found in the first 1000 results from GS, post hoc GS searches were conducted. Original search strategies used for the SR were combined with author names preceded by “author:” and distinct words or phrases from titles, preceded by “intitle:”. If included references were retrieved by this search, they were identified as part of the overall recall of the total number of hits reported. Where combinations of these data elements for the included reference, together with the original search strategies, exceeded 256 characters, the original search strategies were divided into separate searches. Positive hits were confirmed when both separate searches, combined with the article’s metadata, retrieved the item.

When included references were present in GS as *citations only*, this was documented regardless of whether they had been found in the first 1000 search results, in the total search results or as a positive coverage. When included references were found as *citations only*, all citing articles were checked. When the single article citing this included reference was the published review for which the search strategy was first designed, we concluded that the result must have been indexed after the search strategy was originally performed. This included reference was thus not taken into account in the overall coverage of GS. For all three databases, coverage of non-retrieved included references from the inclusion sets was checked thoroughly by searching the databases for author names, distinct words from titles and publication year, using multiple combinations if necessary to ensure no included references were missed.

From these results, overall coverage (number of included references available in the database divided by the total number of included references), recall (number of included references found in the search results for the original search strategies for a database divided by the total number of included references retrieved by all databases together), and precision (number of included references retrieved by a certain database divided by the total number of search results retrieved by that database) of the three databases were calculated. We additionally calculated recall and precision for the first 1000 hits of GS. All data were calculated for the total set of included references (overall values), as well as per review. After we determined which search strategies scored exceptionally well or low on recall in the first 1000 search results in GS, we examined the characteristics of the search strategies (topics and number of search terms) and search results (number of hits and number of included references).

**Fig. 1:**
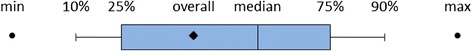
Legend of boxplot figures

We visualized most data in boxplot figures. A general legend can be found in Fig. 1.

## Results

**Fig. 2:**
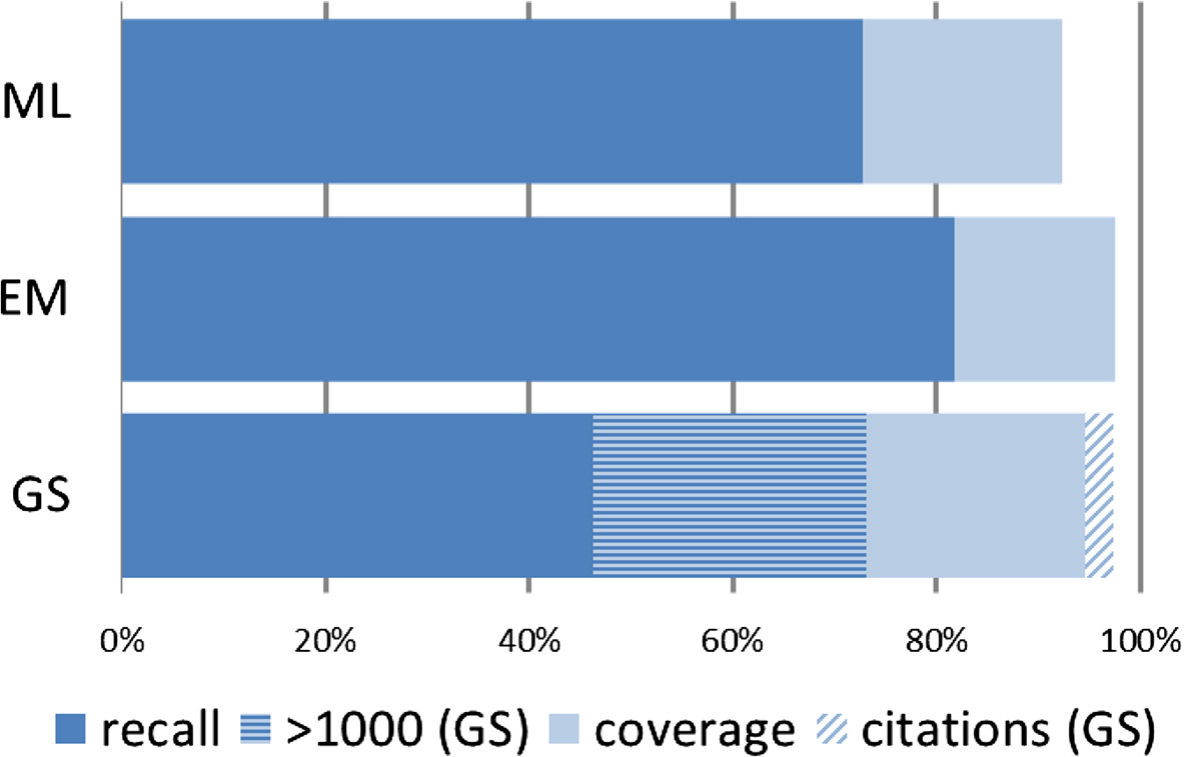
Total coverage and recall of MEDLINE (ML), Embase/MEDLINE (EM), and Google Scholar (GS)

Between May 2013 and August 2015, 520 exhaustive searches designed for SRs by the first author were saved and documented. In August 2015, the reviewers of 120 SRs had screened all search results against their review’s unique inclusion and exclusion criteria. In aggregate, these reviews had included a total of 4795 references. The results for overall recall and coverage of the original 120 search strategies for all three databases for these 4795 included references are summarized in Fig. 2.

### Overall coverage

Overall, GS contained 4708 of the total number of included references (*N* = 4795). However, 179 of these were present as citations only. In 49 of these *citation only* results, the only citing paper in GS was the review based on our search strategy. These 49 search results could not have been covered in GS at the time of the original search. Therefore, overall coverage of the included references was 4659 (97.2 %). In Embase, the percentage of included references found in the database was slightly above GS at 97.5 % while MEDLINE produced 92.3 % of all included references (See Fig. 2).

### Coverage per SR

**Fig. 3:**
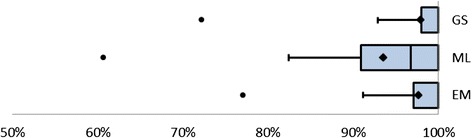
Coverage per SR for GS, MEDLINE, and Embase/MEDLINE

For individual SRs, the percentage of included references present in the three databases varied. For 68 % of all SRs, the coverage of GS was 100 %, Embase contained 100 % of all included references for 63 % of all reviews, compared to 34 % for MEDLINE. For individual SRs, the recall of GS can be as low as 72 %; 77 % was the lowest observation in Embase and 61 % in MEDLINE. See Fig. 3 for a visualization of the coverage per SR.

### Overall recall

In terms of overall recall, Embase/MEDLINE was the most complete, retrieving 3914 of all included references (81.6 %), while MEDLINE alone retrieved 3481 included references (72.6 %). Counting all search results found by the search strategies, GS retrieved 3493 included references (72.8 %). However, only 2224 of those were downloaded with the combined first 1000 search results for the 120 SRs, so the actual recall of GS is much lower at 46.4 % (See Fig. 2).

### Recall per SR

For individual SRs, the percentage of included references present in the first 1000 search results in GS varied by a wide margin. In fifteen SRs, fewer than 25 % of all included references that had been identified through database searches were found in the first 1000 search results of GS, but nine SRs achieved the maximum 100 %.

**Fig. 4:**
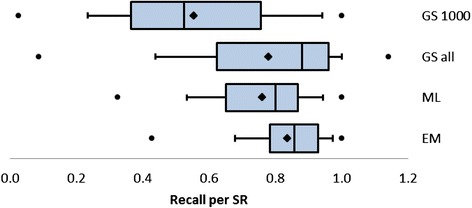
Recall per SR for the first 1000 references in GS, total GS, MEDLINE, and Embase/MEDLINE

Recall fared much better in GS when all search results were taken into account (see Fig. 4). A rate of at least 100 % was reached in 24 SRs (20.0 %). For four SRs, the recall of all search results in GS was even higher than 100 % because GS was able to find included references that had not been found in the traditional databases but were identified via other sources (e.g., reference checking or hand searching). The recall of traditional databases such as Embase and MEDLINE was more consistent of which Embase/MEDLINE performed the best, although its minimum recall was only 43 %.

### Overall precision

**Fig. 5:**
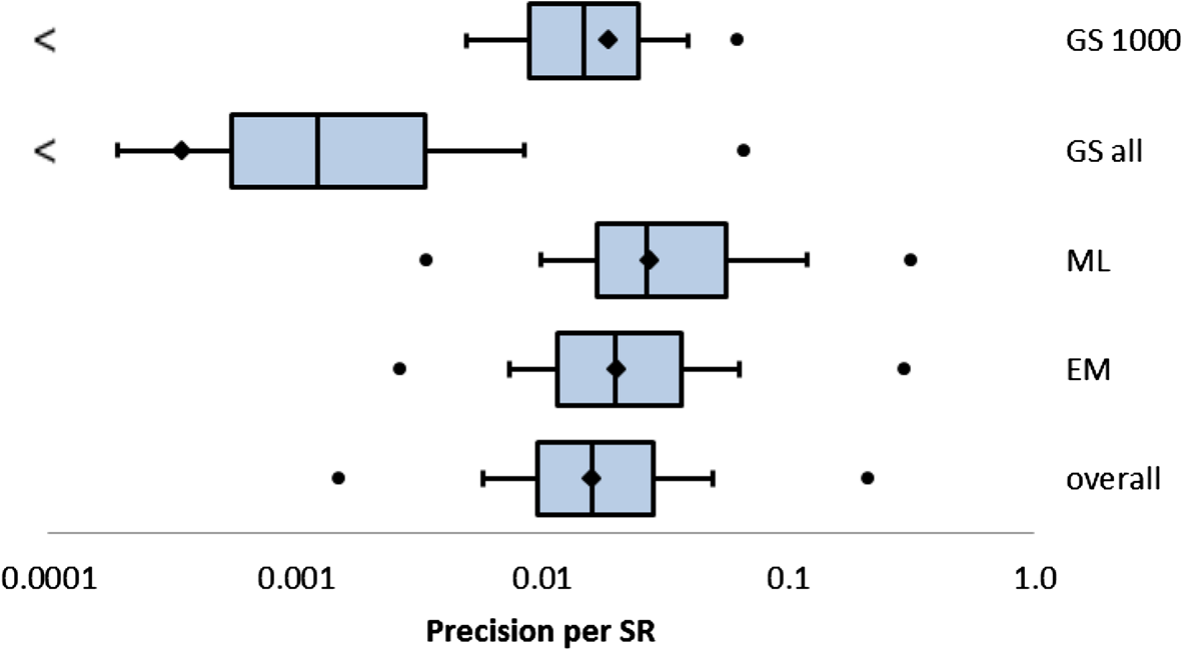
Precision per SR for the first 1000 references in GS, GS, MEDLINE, Embase/MEDLINE, and overall

The total number of search results that were downloaded from GS was 118,509 (in 4 of 120 reviews the number of hits in GS was lower than 1000). These search results together contained 2224 of the included references in the SRs; thus, the overall precision of the first 1000 search results of GS is 1.9 %. The total reported number of search results in GS was 10,092,939, of which 3493 were included references; thus, the overall precision of the complete search results of GS was 0.03 %. The precision of Embase was 3940/192,935 = 2.0 %, and for MEDLINE 3506/126,657 = 2.8 %. These data are visualized within Fig. 5.

### Precision per SR

The precision of GS’ first 1000 search results (1.9 %) did not differ much from the precision as observed in all databases (1.6 %) that were searched in the review process (see Fig. 5). However, the precision of the total set of search results in GS was much lower than that of the other databases (0.03 %).

Why GS scored low is a valid question. Some reasons are discussed below. In some cases (Ahmadi et al. [6], Ambagtsheer (not yet published), Leermakers, Moreira (7)), the first author, together with the reviewer, had not been able to translate a complicated embase.com search strategy into a GS search, due to the lack of proximity operators. Another reason was that the search strategy of Embase was too long for all important search terms to be used in the GS search strategy. In some cases, recall in the traditional databases was possibly higher because of the use of thesaurus terms for a broad topic (such as sexual risk behavior, Legemate, not yet published) or because the fact that topic was very broad which could have resulted in many non-medical references in GS (music in premature infants, Oliai Araghi, not yet published). In other cases, it is unclear why there is such a vast difference between recall in Embase and GS (Bramer [7]). SRs where GS scored exceptionally well often try to answer well-defined topics, such as cashew nut allergy (van der Valk et al. [8]), or platelet-rich plasma injections for tennis elbow (de Vos et al. [9]).

Search strategies for Embase.com, Medline via Ovid, and GS for already published reviews where GS scored exceptionally high or low (as cited in the paragraph above) are shown in the Additional file 1.

## Discussion

GS covers a vast amount of literature but, when excluding *citation only* results first indexed after publication of the reviews used in this research, overall coverage of Embase is slightly higher. Overall recall of GS is not higher than when searching MEDLINE only, and much lower than when searching both MEDLINE and Embase. Since only the first 1000 search results of GS can be used, practical recall of GS is exceptionally low, which makes GS unacceptable as a single database to support the SR. If all search results in GS were made available to users, recall would still be too low for SRs, but reviewer burden would increase due to loss of precision. In fact, none of the observed databases can be used as single databases for SR searching, as the best performing database (Embase/MEDLINE) for individual SRs can result in a recall of less than 50 %. Our observations are similar to recent observations made by Haddaway et al. [10] who compared the recall of GS to that of Web of Science for SRs in the field of environmental science.

Low precision has always been considered a problem in GS [11], but when accounting for actually usable search results (i.e., the first listed 1000), we observed precision to be only slightly lower than the 2.9 % as reported by Sampson et al. in 2011, and comparable to that observed in the other databases [12]. Further, the precision observed in all databases in our study was nearly equal to the practical precision of the first 1000 hits of GS.

The results of this prospective research are for the most part comparable to our previous, retrospective, study. The coverage of GS and recall in MEDLINE are similar to those observed in 2013. However, recall in Google Scholar is much lower for our original searches than for the reconstructed searches in our previous study (45.1 vs. 72 %). That is probably because our search strategies, as they were designed by an experienced information specialist, were optimized to find as many included references as possible in the traditional databases. We translated these search strategies with our best of knowledge into a GS search strategy but were unable to reach a high recall in GS as we had succeeded in the traditional databases.

In SRs, ideally, extended search methods that go beyond traditional databases are used to find included references. Total number of included references is therefore sometimes higher than the number of included references retrieved in the downloaded search results from traditional databases. In evaluating the results of GS search strategies for known items from the included references, some articles did meet the search strategy’s criteria but were not considered relevant enough by GS to be among the first 1000 viewable search results. For some SRs, therefore, the recall of the complete GS search results was higher than 100 %.

The current research could be improved by using search strategies created by multiple independent information specialists at baseline, but we question whether such a change would alter our conclusions. Research on GS for SRs should not focus on whether to use the search tool as a single source but whether it adds value to the search results from other databases. The authors are currently collecting data for a follow-up study that can answer the question whether GS is able to locate included references unidentified by the traditional databases.

## Conclusions

Despite its vast coverage of the scholarly literature, Google Scholar is not sufficient to be used on its own as a single database to support SR searching. The reason for this is not low precision in GS searching, which is comparable to traditional databases. More problematic is GS’ low recall capabilities which are related to the viewable 1000 search results only policy of the search engine. Even if Google Scholar was to allow users to browse beyond the first 1000 search results, its overall recall would still be too low to locate all included references to support the systematic review. We conclude similarly that neither Embase nor MEDLINE on its own is sufficient in retrieving all included references for SRs.

### List of definitions

*Boolean operator*—Set of words (AND, OR, NOT, or proximity operators) used as conjunctions to combine or exclude keywords in a search strategy.

*Citation only*—Search result retrieved by Google Scholar solely because another article cited it in its list of references.

*Coverage*—Number of included references available in a certain database divided by the number of relevant articles included in a systematic review.

*Included reference*—A specific article that, after consideration of inclusion and exclusion criteria, is included by review authors in their systematic review.

*Librarian-mediated searches*—Searches that are designed by (medical) librarians or information specialists in close accordance with researchers’ information needs and research goals.

*Optimization of search strategies*—Improving recall or precision of search strategies by adding or dropping search terms or key concepts.

*Practical precision in Google Scholar*—Percentage of included hits in the first 1000 search results of Google Scholar.

*Precision*—Number of included references retrieved divided by the total number of articles retrieved.

*Proximity operator*—A special kind of Boolean operator used to search for occurrences of words adjacent to or within a certain number of words from another word (or group of words).

*Recall*—Number of included references retrieved by one database divided by the number of included references retrieved by all databases together.

*Reviewer*—A person requesting a librarian-mediated search, for a systematic review, who is responsible for reviewing search yield or results and determining which references meet predetermined inclusion criteria.

*Search result*—The references (or the number thereof) provided by a certain database that fulfill the criteria of a search strategy.

*Search strategy*—A sequence of search terms (thesaurus terms and free text) combined with Boolean operators designed to find relevant search results in a certain database.

*Single-line search strategy*—Search strategies consisting of one line of search terms combined with Boolean operators and parentheses, as opposed to multi-line search strategies, which combine search results from multiple record sets.

## Additional file

Additional file 1: Search strategies for published systematic reviews where the recall of Google Scholar was exeptionally good or poor. (DOCX 23 kb)

## Abbreviations

EM: Embase/MEDLINE combination
GS: Google Scholar
ML: MEDLINE
SR: systematic review
