# Supplementary material for: Comparing the coverage, recall, and precision of searches for 120 systematic reviews in Embase, MEDLINE, and Google Scholar: a prospective study
Source: Syst Rev. 2016 Mar 1;5:39. doi: 10.1186/s13643-016-0215-7 (PMC4772334; doi:10.1186/s13643-016-0215-7)
Supplement: Additional file 1: — Search strategies for published systematic reviews where the recall of Google Scholar was exeptionally good or poor. (DOCX 23 kb) [file 13643_2016_215_MOESM1_ESM.docx]

**Bramer et al - Comparing the coverage, recall and precision of searches for 120 systematic reviews**

**Supplementary material – Search strategies for published systematic reviews where the recall of Google Scholar was exeptionally good or poor.**

**Ahmadi, Lafranca**

Ahmadi AR, Lafranca JA, Claessens LA, Imamdi RM, IJzermans, JN, Betjes MG, et al. Shifting paradigms in eligibility criteria for live kidney donation: a systematic review. Kidney Int. 2015 Jan;87(1):31-45.

**Total number of included references: 115**

**Number of included references found in the databases: 106**

**Embase : 1530 hits**

**101 references found**

**Recall: 95%**

**Precision: 6.6%**

(('living donor'/de OR (((living OR live OR laparoscop*) NEAR/3 (donor* OR donat* OR transplant*))):ab,ti) AND (('kidney transplantation'/exp NOT 'cadaver kidney'/de) OR kidney/de OR 'kidney donor'/de OR nephrectomy/de OR (kidney* OR renal OR nephrectom*):ab,ti)) AND (comorbidity/de OR comorbidit*:ab,ti OR (age/de OR (((age OR old*) NEAR/3 (factor* OR donor* OR donat*)) OR aged* OR elderl* OR senior* OR 'older age' OR 'older than' OR ((over OR above OR past) NEXT/2 (year* OR age)) OR (donor* NEXT/1 over) OR 'advanced age'):ab,ti) OR (('body mass'/de OR 'body weight'/de OR 'body weight disorder'/de OR 'weight gain'/de OR obesity/exp OR (mass OR weight OR BMI OR quetelet OR obes* OR overweight*):ab,ti) OR (hypertension/exp OR (hypertens* OR 'blood pressure'):ab,ti) OR ('congenital blood vessel malformation'/de OR 'arteriovenous malformation'/de OR ((arter* OR vein* OR venous* OR vessel* OR vascul*) NEAR/3 (anomal* OR malformat* OR multipl*)):ab,ti) OR nullipara/de OR (nullipar*):ab,ti OR juvenile/de OR (juvenile OR minors):ab,ti)) AND ('donor selection'/de OR ((donor* NEAR/3 select*) OR criteri* OR ((after OR post) NEXT/3 (donat* OR nephrectom*)) OR postdonat* OR ((donor* OR nephrectom*) NEAR/6 ('follow up' OR safet* OR outcome* OR risk* OR complication* OR morbid* OR mortal* OR postoperat* OR intraoperat* OR postsurg* OR intrasurg* OR 'post operative' OR 'intra operative' OR 'post surgery' OR 'intra surgery' OR hospitalization OR hospitalization OR deteriorat*))):ab,ti)

**Medline OvidSP : 1022 hits**

**66 references found**

**Recall: 62%**

**Precision: 6.5%**

("Living Donors"/ OR (((living OR live OR laparoscop*) ADJ3 (donor* OR donat* OR transplant*))).ab,ti.) AND ("kidney transplantation"/ OR exp kidney/ OR nephrectomy/ OR (kidney* OR renal OR nephrectom*).ab,ti.) AND (("kidney transplantation"/ NOT "cadaver kidney"/) OR kidney/ OR "kidney donor"/ OR nephrectomy/ OR (kidney* OR renal OR nephrectom*).ab,ti.) AND (comorbidity/ OR comorbidit*.ab,ti. OR ("Age Factors"/ OR (((age OR old*) ADJ3 (factor* OR donor* OR donat*)) OR aged* OR elderl* OR senior* OR "older age" OR "older than" OR ((over OR above OR past) ADJ2 (year* OR age)) OR (donor* ADJ over) OR "advanced age").ab,ti.) OR ("Body Mass Index"/ OR exp "body weight"/ OR exp obesity/ OR (mass OR weight OR BMI OR quetelet OR obes* OR overweight*).ab,ti.) OR (exp hypertension/ OR (hypertens* OR "blood pressure").ab,ti.) OR (exp "Arteriovenous Malformations"/ OR ((arter* OR vein* OR venous* OR vessel* OR vascul*) ADJ3 (anomal* OR malformat* OR multipl*)).ab,ti.) OR Parity/ OR (nullipar*).ab,ti. OR minors/ OR (juvenile OR minors).ab,ti.) AND ("donor selection"/ OR ((donor* ADJ3 select*) OR criteri* OR ((after OR post) ADJ3 (donat* OR nephrectom*)) OR postdonat* OR ((donor* OR nephrectom*) ADJ6 ("follow up" OR safet* OR outcome* OR risk* OR complication* OR morbid* OR mortal* OR postoperat* OR intraoperat* OR postsurg* OR intrasurg* OR post operat* OR intra operat* OR post surg* OR intra surg* OR hospitalization OR hospitalization OR deteriorat*))).ab,ti.)

**Google scholar**

**12 references found**

**Recall: 11.3%**

**Precision: 1.2%**

"living|live|laparoscopic kidney|renal donor|donation|transplantation" comorbidity|elderly|body-weight|obesity|BMI|overweight|hypertension "donor selection"|"after|post donation"|postdonation|"donor safety|outcome|complications|morbidity"

**Bramer**

Bramer WM. Evaluation of instructive texts on searching medical databases. J Med Libr Assoc. 2015 Oct;103(4):208-9.

**Total number of included references: 49**

**Number of included references found in the databases: 38**

**Embase : 2199 hits**

**33 references found**

**Recall: 87%**

**Precision: 1.5%**

(('bibliographic database'/exp/mj AND 'information retrieval'/exp/mj) OR (((information OR literature* OR article* OR reference* OR database* OR PubMed OR medline OR embase* OR stud*) NEAR/3 (retriev* OR search* OR discover* OR locate* OR find* OR seek*)) OR ((search* OR seek*) NEAR/3 (method* OR optim* OR skill* OR improv* OR expert* OR librar* OR comprehens* OR enough* OR quick* OR efficien* OR effect* OR strateg* OR sensitiv* OR specific*))):ti) AND (standardization/exp OR 'total quality management'/exp OR 'quality control'/exp OR error/exp OR library/exp OR 'professional competence'/exp OR teaching/exp OR 'selection bias'/exp OR (standard OR standards OR standardizat* OR standardisat* OR qualit* OR error* OR 'how to' OR handbook* OR guideline* OR librar* OR competen* OR teach*):ab,ti) AND [english]/lim AND [1995-2015]/py NOT ([animals]/lim NOT [humans]/lim)

**Medline : 2314 hits**

**25 references found**

**Recall: 66%**

**Precision: 1.1%**

((exp *Databases, Bibliographic/ AND exp *Information Storage and Retrieval/) OR (((information OR literature* OR article* OR reference* OR database* OR PubMed OR medline OR embase* OR stud*) ADJ3 (retriev* OR search* OR discover* OR locate* OR find* OR seek*)) OR ((search* OR seek*) ADJ3 (method* OR optim* OR skill* OR improv* OR expert* OR librar* OR comprehens* OR enough* OR quick* OR efficien* OR effect* OR strateg* OR sensitiv* OR specific*))).ti.) AND (standards.xs. OR total quality management/ OR quality control/ OR Selection Bias/ OR Research Design/ OR exp Libraries/ OR professional competence/ OR teaching/ OR (standard OR standards OR standardizat* OR standardisat* OR qualit* OR error* OR how to OR handbook* OR guideline* OR librar* OR competen* OR teach*).ab,ti.) AND english.la. NOT (exp animals/ NOT humans/)

**Google Scholar**

**1 reference found**

**Recall: 2.6%**

**Precision: 0.1%**

"information|literature retrieval|searching"|"searching|search method|methods|optimization|skill|skills|improvement"|"expert|librarian|comprehensive search" standard|standards|standardization|quality medical|"systematic review|reviews"|clinical

**Leermakers, Moreira**

Leermakers ET, Moreira EM, Kiefte-de Jong JC, Darweesh SK, Visser T, Voortman T, et al. Effects of choline on health across the life course: a systematic review. Nutr Rev. 2015 Aug;73(8):500-22.

**Total number of included references: 50**

**Number of included references found in the databases: 45**

**Embase : 3073 hits**

**30 references found**

**Recall: 67%**

**Precision: 1.0%**

(Choline/de OR 'choline alfoscerate'/de OR 'choline bitartrate'/de OR citicoline/de OR phosphorylcholine/de OR phosphatidylcholine/de OR ((beta NEXT/1 cholin*) OR 'alpha lecithin' OR amonita OR 'b cholin' OR bilineurine OR biocholine OR biocolina OR 'brassel 1000' OR bursine OR ceraxon OR cholin OR choline OR cholinephosphate OR cidifos OR cidiphos OR citicholine OR citicolin* OR cyticholin* OR cytocholine OR diacylglycerophosphocholine OR diacylglycerophosphorylcholine OR diacylphosphatidylcholine OR diphosphocholine OR 'egg lecithin' OR fagine OR fosfatidylcholine OR fosfolutein OR glycerophosphatidylcholine OR glycerophosphocholine OR glycerylphosphorylcholine OR granulestin OR hepacholine OR kelecin OR laevocholine OR lecithin* OR lecithol OR levocholine OR lipotril OR luridine OR maxicholine OR nicholin OR phosphatidylcholine* OR phosphocholine OR phospholipon OR phosphorylcholine OR rexort OR sauran OR sincaline OR sinkron OR sintoclar OR somazina OR topcithin OR urocholine OR vegelecithin OR 'Vitamine B4'):ab,ti) AND (((('cardiovascular disease'/exp NOT ('cardiovascular disease'/exp/dm_cn OR 'congenital disorder'/exp)) OR ((cardiovascular OR cardiac OR heart OR vascular OR cardiometabolic) NOT congenital):ab,ti) OR 'non insulin dependent diabetes mellitus'/de OR ((diabetes NEAR/6 ('type 2' OR 'type ii' OR 'non insulin' OR noninsulin)) OR ((glucose OR insulin) NEAR/3 (level* OR concentration OR plasma OR blood OR serum OR metabolism OR tolerance OR intolerance OR sensitivit* OR insensitivity* OR resistance OR homeosta*)) ):ab,ti) OR (hypertension/exp OR (('blood pressure') OR hypertensi* OR ((cholesterol OR LDL* OR HDL* OR triglyceride* OR lipoprotein* OR lipid*) NEAR/3 (plasma OR blood OR serum OR level* OR profile*)) OR hyperlip* OR dyslip*):ab,ti) OR (obesity/exp OR 'body mass'/de OR 'body fat'/de OR 'waist circumference'/de OR 'waist hip ratio'/de 'Metabolic Syndrome X'/de OR 'dual energy X ray absorptiometry'/de OR (obesity OR obese OR 'over weight' OR overweight OR adiposity OR dexa OR dxa OR 'dual energy X ray absorptiometry' OR 'dual x ray absorptiometry' OR ‘metabolic syndrome’ OR ‘body mass index’ OR BMI OR Quetelet OR (body NEXT/1 (composition* OR fat* OR weight*)) OR 'ponderal index' OR (weight NEXT/1 gain*) OR ‘abdominal fat’ OR (fat NEAR/3 (mass OR percentage*) OR skinfold* OR (waist NEAR/3 (hip OR circumference*))):ab,ti)) OR (autacoid/de OR chemokine/exp OR 'prostaglandin derivative'/de OR 'C reactive protein '/de OR (autacoid* OR chemokine* OR prostaglandin* OR ((inflammat*) NEAR/3 (marker* OR mediator*)) OR 'C reactive protein' OR 'creactive protein' OR crp OR 'c reaction protein'):ab,ti) OR ('anthropometric parameters'/de OR 'body height'/de OR 'body size'/de OR 'head circumference'/de (((body) NEAR/3 (height OR size)) OR 'head circumference' OR (height NEAR/3 age)):ab,ti) OR ('respiratory tract disease'/exp OR 'respiratory function'/exp OR 'lung function test'/exp OR 'cystic fibrosis'/de OR (((respirat* OR breath* OR pulmonar* OR lung* OR airway* OR bronchopulmon*) NEAR/3 (disease* OR function* OR disorder* OR obstruct*)) OR COPD OR bronchitis):ab,ti) OR ('mental disease'/exp OR Epilepsy/exp OR cognition/exp OR 'mental function'/de OR 'brain function'/de OR memory/de OR (((cognit* OR learn* OR brain* OR neurolog* OR mental*) NEAR/3 (disorder* OR disease* OR function* OR develop* OR impair*)) OR memor* OR dyslex* OR (Attention NEAR/3 Defic*) OR adhd OR epilep* OR cognit* OR dement*):ab,ti) OR ('nonalcoholic fatty liver'/de OR ((nonalcoholic OR 'non alcoholic' ) NEAR/3 ('fatty liver' OR steatohepatitis)):ab,ti)) AND (Epidemiology/exp OR 'cohort analysis'/de OR 'prospective study'/de OR 'follow up'/de OR 'longitudinal study'/de OR 'retrospective study'/de OR 'case control study'/de OR 'intervention study'/de OR 'clinical study'/de OR 'clinical trial'/exp OR (((Hazard OR odds OR risk*) NEXT/1 Ratio*) OR ((Prospectiv* OR Populat* OR Observat* OR Retrospect* OR intervent* OR clinical) NEXT/1 (stud* OR trial*)) OR (case* NEAR/3 control*) OR (Cross NEXT/1 section*)):ab,ti) NOT ([animals]/lim NOT [humans]/lim)

**Medline : 1570 hits**

**29 references found**

**Recall: 64%**

**Precision: 1.8%**

(Choline/ OR Glycerylphosphorylcholine/ OR exp Phosphatidylcholines/ OR (beta cholin* OR "alpha lecithin" OR amonita OR "b cholin" OR bilineurine OR biocholine OR biocolina OR "brassel 1000" OR bursine OR ceraxon OR cholin OR choline OR cholinephosphate OR cidifos OR cidiphos OR citicholine OR citicolin* OR cyticholin* OR cytocholine OR diacylglycerophosphocholine OR diacylglycerophosphorylcholine OR diacylphosphatidylcholine OR diphosphocholine OR "egg lecithin" OR fagine OR fosfatidylcholine OR fosfolutein OR glycerophosphatidylcholine OR glycerophosphocholine OR glycerylphosphorylcholine OR granulestin OR hepacholine OR kelecin OR laevocholine OR lecithin* OR lecithol OR levocholine OR lipotril OR luridine OR maxicholine OR nicholin OR phosphatidylcholine* OR phosphocholine OR phospholipon OR phosphorylcholine OR rexort OR sauran OR sincaline OR sinkron OR sintoclar OR somazina OR topcithin OR urocholine OR vegelecithin OR "Vitamine B4").ab,ti.) AND ((((exp "cardiovascular diseases"/ NOT (exp cardiovascular diseases/cn OR exp "Congenital Abnormalities"/)) OR ((cardiovascular OR cardiac OR heart OR vascular OR cardiometabolic) NOT congenital).ab,ti.) OR "Diabetes Mellitus, Type 1"/ OR ((diabetes ADJ6 ("type 2" OR "type ii" OR "non insulin" OR noninsulin)) OR ((glucose OR insulin) ADJ3 (level* OR concentration OR plasma OR blood OR serum OR metabolism OR tolerance OR intolerance OR sensitivit* OR insensitivity* OR resistance OR homeosta*)) ).ab,ti.) OR (exp hypertension/ OR (("blood pressure") OR hypertensi* OR ((cholesterol OR LDL* OR HDL* OR triglyceride* OR lipoprotein* OR lipid*) ADJ3 (plasma OR blood OR serum OR level* OR profile*)) OR hyperlip* OR dyslip*).ab,ti.) OR (exp obesity/ OR "Body Mass Index"/ OR exp "Body Fat Distribution"/ OR exp "Adipose Tissue"/ OR "waist circumference"/ OR "Waist-Hip Ratio"/ OR "Metabolic Syndrome X"/ OR "Absorptiometry, Photon"/ OR (obesity OR obese OR "over weight" OR overweight OR adiposity OR dexa OR dxa OR "dual energy X ray absorptiometry" OR "dual x ray absorptiometry" OR "metabolic syndrome" OR "body mass index" OR BMI OR Quetelet OR (body ADJ (composition* OR fat* OR weight*)) OR "ponderal index" OR (weight ADJ gain*) OR "abdominal fat" OR (fat ADJ3 (mass OR percentage*)) OR skinfold* OR (waist ADJ3 (hip OR circumference*))).ab,ti.) OR (exp autacoids/ OR exp chemokines/ OR "C-Reactive Protein"/ OR (autacoid* OR chemokine* OR prostaglandin* OR (inflammat* ADJ3 (marker* OR mediator*)) OR "C reactive protein" OR "creactive protein" OR crp OR "c reaction protein").ab,ti.) OR ("Body Weights and Measures"/ OR "Body Constitution"/ OR "Skinfold Thickness"/ OR exp "body size"/ OR (((body) ADJ3 (height OR size)) OR head circumference OR (height ADJ3 age)).ab,ti.) OR (exp "respiratory tract diseases"/ OR exp "Respiratory Physiological Phenomena"/ OR exp "Respiratory Function Tests"/ OR "cystic fibrosis"/ OR (((respirat* OR breath* OR pulmonar* OR lung* OR airway* OR bronchopulmon*) ADJ3 (disease* OR function* OR disorder* OR obstruct*)) OR COPD OR bronchitis).ab,ti.) OR (exp "Mental Disorders"/ OR exp Epilepsy/ OR exp cognition/ OR exp "Mental Processes"/ OR exp memory/ OR exp "Memory Disorders"/ OR (((cognit* OR learn* OR brain* OR neurolog* OR mental*) ADJ3 (disorder* OR disease* OR function* OR develop* OR impair*)) OR memor* OR dyslex* OR (Attention ADJ3 Defic*) OR adhd OR epilep* OR cognit* OR dement*).ab,ti.) OR (((nonalcoholic OR 'non alcoholic' ) ADJ3 ('fatty liver' OR steatohepatitis)).ab,ti.)) AND (exp Epidemiologic Studies/ OR "Intervention Studies"/ OR "clinical trial".pt. OR (((Hazard OR odds OR risk*) ADJ Ratio*) OR ((Prospectiv* OR Populat* OR Observat* OR Retrospect* OR intervent* OR clinical) ADJ (stud* trial*)) OR (case* ADJ3 control*) OR (Cross ADJ section*)).ab,ti.) NOT (exp animals/ NOT humans/)

**Google Scholar**

**9 references found**

**Recall: 20%**

**Precision: 0.9%**

Choline cardiac|heart|diabetes |hypertension|obesity|"body fat|mass|weight|height|size"|overweight|adiposity|COPD|bronchitis|"brain|mental disease|function"|Epilepsy|cognition|memory|adhd cohort|prospective|"follow up"|longitudinal|retrospective

**van der Valk, Dubois**

van der Valk JP, Dubois AE, Gerth van Wijk R, Wichers HJ, de Jong NW. Systematic review on cashew nut allergy. Allergy. 2014 Jun;69(6):692-8.

**Total number of included references: 16**

**Number of included references found in the databases: 16**

**Embase : 352 hits**

**15 references found**

**Recall: 94%**

**Precision: 4.3%**

(Anacardiaceae/de OR Anacardium/exp OR 'anacardic acid'/de OR (Anacardi* OR cashew*):ab,ti) AND (hypersensitivity/exp OR 'occupational disease'/exp OR 'occupational exposure'/de OR 'occupational health'/de OR 'occupational hazard'/de OR sensitization/exp OR 'industrial worker'/de OR 'immune function test'/exp OR 'immune response'/exp OR provocation/de OR allergen/exp OR 'skin irritation'/exp OR (hypersens* OR (hyper NEXT/1 sens*) OR allerg* OR occupation* OR dermatitis OR phytodermatitis OR eczem* OR anaphyla* OR sensitiz* OR cosensitiz* OR ((industr* OR factor* OR cashew*) NEAR/3 (worker* OR employee* OR personnel*)) OR provocat* OR ((patch OR prick ) NEXT/1 test*) OR (immun* NEAR/3 (respons* OR react*)) OR immunoreact* OR irritat* OR hazard*):ab,ti)

**Medline : 171 hits**

**14 references found**

**Recall: 88%**

**Precision: 8.2%**

(Anacardiaceae/ OR Anacardium/ OR "Anacardic Acids"/ OR (Anacardi* OR cashew*).ab,ti.) AND (exp hypersensitivity/ OR exp "occupational diseases"/ OR exp "occupational exposure"/ OR "occupational health"/ OR "Skin Irritancy Tests"/ OR exp allergens/ OR (hypersens* OR (hyper ADJ sens*) OR allerg* OR occupation* OR dermatitis OR phytodermatitis OR eczem* OR anaphyla* OR sensitiz* OR cosensitiz* OR ((industr* OR factor* OR cashew*) ADJ3 (worker* OR employee* OR personnel*)) OR provocat* OR ((patch OR prick ) ADJ test*) OR (immun* ADJ3 (respons* OR react*)) OR immunoreact* OR irritat* OR hazard*).ab,ti.)

**Google Scholar**

**16 references found**

**Recall: 100%**

**Precision: 1.6%**

cashew hypersensitivity|"hyper sensitivity"|allergy|allergic|occupational|dermatitis|eczema|anaphylaxis|anaphylactic|sensitization|"industrial|factory worker|employee|personnel"|provocation|"patch|prick test"|irritation|hazard

**de Vos, Windt**

de Vos RJ, Windt J, Weir A. Strong evidence against platelet-rich plasma injections for chronic lateral epicondylar tendinopathy: a systematic review. Br J Sports Med. 2014 Jun;48(12):952-6.

**Total number of included references: 7**

**Number of included references found in the databases: 7**

**Embase : 168 hits**

**6 references found**

**Recall: 86%**

**Precision: 3.6%**

('tennis elbow'/de OR ((elbow/de OR 'elbow disease'/exp OR 'elbow injury'/de OR (elbow*):ab,ti) AND ('tendinitis'/de OR 'tendon injury'/exp OR (tendinos* OR tendinit* OR tendinopath* OR ((tendon*) NEAR/3 (injur* OR trauma*))):ab,ti)) OR ((tennis NEXT/1 (elbow* OR arm*)) OR ((epicondyl* OR bursitis) NEAR/3 (humer* OR lateral* OR radiohumer*)) OR (chronic* NEAR/3 tendinopath*)):ab,ti) AND ('thrombocyte rich plasma'/de OR ((plasma/de) AND (injection/de)) OR 'blood autotransfusion'/de OR (((thrombocyte* OR platelet*) NEAR/3 (plasma)) OR PRP OR autotransfus* OR (autologous NEAR/3 blood )):ab,ti)

**Medline : 97 hits**

**5 references found**

**Recall: 71%**

**Precision: 5.2%**

("tennis elbow"/ OR ((elbow/ OR "elbow disease"/ OR "elbow injury"/ OR (elbow*).ab,ti.) AND (exp "tendon injuries"/ OR (tendinos* OR tendinit* OR tendinopath* OR ((tendon*) ADJ3 (injur* OR trauma*))).ab,ti.)) OR ((tennis ADJ (elbow* OR arm*)) OR ((epicondyl* OR bursitis) ADJ3 (humer* OR lateral* OR radiohumer*)) OR (chronic* ADJ3 tendinopath*)).ab,ti.) AND ("Platelet-Rich Plasma"/ OR ((plasma/) AND (exp injections/)) OR "Blood Transfusion, Autologous"/ OR (((thrombocyte* OR platelet*) ADJ3 (plasma)) OR PRP OR autotransfus* OR (autologous ADJ3 blood )).ab,ti.)

**Google Scholar**

**7 references found**

**Recall: 100%**

**Precision: 0.7%**

("tennis (elbow|arm)"|"(humeral|lateral|radiohumeral)(epicondylar|bursitis)") "(thrombocyte|platelet) rich plasma"|autotransfusion|"autologous blood")

*The GS search contains parentheses. Later we learned that parentheses are unnecessary in GS searches. However we present the search as it was executed for this review.*
